# Non-surgical spinal decompression therapy: does the scientific literature support efficacy claims made in the advertising media?

**DOI:** 10.1186/1746-1340-15-7

**Published:** 2007-05-18

**Authors:** Dwain M Daniel

**Affiliations:** 1 Parker Research Institute, Parker College of Chiropractic, Dallas Texas, USA

## Abstract

**Background:**

Traction therapy has been utilized in the treatment of low back pain for decades. The most recent incarnation of traction therapy is non-surgical spinal decompression therapy which can cost over $100,000. This form of therapy has been heavily marketed to manual therapy professions and subsequently to the consumer. The purpose of this paper is to initiate a debate pertaining to the relationship between marketing claims and the scientific literature on non-surgical spinal decompression.

**Discussion:**

Only one small randomized controlled trial and several lower level efficacy studies have been performed on spinal decompression therapy. In general the quality of these studies is questionable. Many of the studies were performed using the VAX-D^® ^unit which places the patient in a prone position. Often companies utilize this research for their marketing although their units place the patient in the supine position.

**Summary:**

Only limited evidence is available to warrant the routine use of non-surgical spinal decompression, particularly when many other well investigated, less expensive alternatives are available.

## Background

Traction as a therapeutic intervention in the treatment of low back pain has existed for many years. Its use has progressed from simple static traction to intermittent motorized traction. A recent systematic review found only seven randomized controlled trials for intermittent motorized traction and six reported no difference in outcomes between the traction groups and the control groups [1]. The most recent incarnation of traction has been a form of intermittent motorized traction commonly referred to as spinal decompression therapy. Developers and manufacturers of the equipment along with clinicians often consider it to be a unique form of traction.

A perusal of any trade publication aimed at manual therapy professions will demonstrate intense marketing programs extolling the virtues of this new technology. An 86% success rate is claimed by many manufacturers and passed on to the consumer through individual practitioner's advertising. A recent limited online poll published in a chiropractic trade magazine stated that 38% of doctors of chiropractic are using the technology in their offices [2]. According to the Job Analysis of Chiropractic the presence of traction in the chiropractor's office has risen from 73.2% in 1991 to 80.6% in 2003 [3], which represents as many as 5,000 new traction units among chiropractors. With units priced from $9,000 to well over $100,000 each, spinal decompression is obviously a significant financial decision for the individual practitioner.

Several papers relating to intermittent and static traction have been published. The purpose of this paper is to open a debate on the efficacy of spinal decompression therapy, defined as motorized traction utilizing variable force, variable traction/relaxation times and in some units, variable angles of pull.

Literature searches were performed in Medline, CINAHL and MANTIS databases from January 1990 through September 2006. Search terms included decompression therapy, traction, treatment outcome, outcome assessment and evaluation studies. Additionally, keyword searches were performed using brand names of specific manufacturers. Additional material was gathered from the research sections of manufacturer web sites and hand searches. Care was taken to insure research quoted on web sites was from peer reviewed scientific journals. It was the original intent of the author to perform a traditional systematic review; that is to search the scientific literature, review the available clinical trials, grade the evidence and finally present the findings. In this case such an effort was not necessary. Only 1 randomized controlled trial, 1 clinical trial, 1 case series and 7 other papers were located. With the exception of a study pertaining to protocols and procedures, these studies will be individually reviewed.

## Discussion

### A prospective randomized controlled study of VAX-D and TENS for the treatment of chronic low back pain [4]

The single randomized controlled trial of spinal decompression therapy compared the VAX-D^® ^unit to transcuteanous electrical nerve stimulation (TENS) for the treatment of chronic low back pain. Subjects were recruited through advertisement and had chronic low back pain of more than 3 months duration with associated leg pain. Disc protrusion or herniation confirmed by CT or MRI was also required. Average duration of pain in the study population was 7.3 years and average age was 42 years old. This study enrolled 44 patients and 40 completed the study. Patients were randomized in sequential order to their appropriate group. Outcome measures were the 10 centimeter visual analog pain scale (VAS) and a disability scale. The disability scale rated the subject's ability to perform their most affected activity on a 0 to 4 scale, with 4 being "can do without limitation". Treatments consisted of 30 minute sessions, five times per week for four weeks followed by weekly sessions for 4 weeks. The control group received TENS for 30 minutes daily for 20 days followed by weekly treatment for 4 weeks. Both groups were able to take anti-inflammatory and non-narcotic pain relievers as needed. Success of treatment was defined by 50% improvement in VAS and any improvement in disability. At the conclusion of the study 13 out of 19 (68.4%) of the treatment group showed improvement while 0 of 21 for the TENS group. At the six month follow-up 7 of the original 19 subjects (36.8%) in the treatment group showed sustained improvement.

#### Study limitations

This study utilized a small sample, did not provide power calculations and may have been underpowered. In a review performed by the Evidence Based Practice Group it was noted that the sequential randomization and statistical analysis used in this study severely limited the effectiveness of randomization [5]. Lack of blinding could have had a significant impact on the outcome as no placebo effect was noted. The control group actually suffered degradation of their symptoms at the conclusion of the study making statistically significant improvement easier to achieve. Although a six month follow-up was reported for the treatment group, it was not reported for the control group.

### Decompression, reduction, and stabilization of the lumbar spine: a cost effective treatment for lumbosacral pain [6]

A clinical trial comparing intermittent motorized traction to spinal decompression (DRS System^®^) was performed and reported in 1997. Twenty-seven men and twelve women were enrolled in the study and randomized to their appropriate group. Twenty-three had ruptured discs confirmed by MRI and 35 had sciatic radiation. Duration of symptoms was less than one year. Sixteen subjects had facet arthrosis with symptoms from one to 20 years. Subjects were blinded to treatment. In addition to the primary interventions, subjects received ice treatments, electric stimulation, and home use of TENS and three sessions with an exercise specialist. The authors state 86% of ruptured disc patients had "good or excellent" results using decompression therapy compared to 55% for traction subjects. Facet arthrosis patients had similar results with 75% improved with decompression therapy compared to 50% for traction.

#### Study limitations

Clearly the most obvious shortcoming of this study is the use of descriptive statistics to report outcomes. No calculations were reported to determine if the improvements in the treatment group were statistically significant compared to the control group. Additionally the methods to determine outcomes were not described. The authors merely stated that excellent = 90 to 100% improved, good = 50 to 89% improved and poor = < 50% improved. What constituted improvement was not discussed.

### Vertebral axial decompression therapy for pain associated with herniated or degenerated discs or facet syndrome: an outcome study [7]

A case series was performed that included 778 cases of low back pain patients that had disc dysfunction or facet syndrome confirmed by diagnostic imaging. Average duration of pain was 4 months or more in 83% of cases. Outcome measures were a 5 point pain scale and self assessment of mobility and ability to walk and sit. Patients were treated with the VAX-D unit and other concurrent, unspecified modalities and medications. Using a reduction in pain scores to 0 or 1 on a 5 point scale was considered a successful outcome. This study claimed a 71% success rate.

#### Study limitations

Although this is a large case series study, it cannot nor does it attempt to determine if the treatment is more effective than a placebo or other available treatments. Concurrent use of other modalities and medicine confound the outcomes since it is unknown which treatment or combination of treatments may have been responsible for the positive response.

### Long-term effect analysis of IDD therapy in low back pain: a retrospective clinical pilot study [8]

A retrospective case series of 33 patients was performed utilizing the Intervertebral Differential Dynamics (IDD) ^® ^unit. The inclusion criteria were simply low back pain. The average age of participants was 73.4 years and the average number of treatment sessions completed was 19. The primary outcome measure was the numeric pain scale (0 representing no pain and 10 representing worst pain). Of the 24 patients completing the study the mean improvement in pain scores from first to last session was 4.46 (p < 0.01) and at the 1 year follow-up 5.23 (p < 0.01). Overall the authors claimed a 76% decrease in pain at the one year follow-up.

#### Study limitations

This is a smaller retrospective study. It is, as is the last study discussed, preliminary in nature. It cannot be used to determine treatment efficacy compared to another treatment or placebo.

### Efficacy of VAX-D on chronic low back pain: Study of dosage regimen [9]

This study compared the effect of 10 treatment sessions to 20 treatment sessions on the VAX-D^® ^decompression unit. One hundred and forty-two consecutive patients with chronic low back pain were treated and evaluated in this study. The visual analog pain scale and activities of daily living were used as outcome measure. Ninety-one patients received 10 sessions of treatment and the remainder received 20 sessions. Improvement of the 20 session group was statistically significant over the 10 treatment group (p < 0.0001).

#### Study limitations

This study was designed with a single purpose, to measure dose response. It cannot address efficacy. The patients in this study were not randomized. Controls were minimal. The demographics of the individuals in the 10 treatment group were not compared to the individuals in the 20 treatment group; consequently it is difficult to establish whether the characteristics of the two groups were similar. These factors weaken the value of the study even for the purposes of dose response.

### Dermatomal somatosensory evoked potential demonstration of nerve root decompression after VAX-D therapy [10]

This case series was performed with 7 subjects to measure the effect of VAX-D^® ^therapy on dermatosomal somatosensory evoked potentials (DSSEP) [10]. All patients had had documented L5/S1 disc herniations. All patients showed improvement in DSSEP's in the ipsilateral or contralateral leg. Two patients showed worsening of DSSEP's in the symptomatic leg although both experienced improvements in symptomology. Overall the authors state that all subjects had at least a 50% improvement in radicular pain and back pain with 3 becoming asymptomatic.

#### Study limitations

The use of DSSEP as a valid outcome measure must be questioned when two of 7 subjects showed worsening of DSSEP's in the symptomatic leg although symptomology improved. Follow-up was not performed on these subjects so it cannot be determined if the effect of treatment was lasting or transient.

### Effects of vertebral axial decompression on intradiscal pressure [11]

This study measured intradiscal pressure of subjects while undergoing decompression therapy on a VAX-D ^® ^therapy unit. Five subjects were selected, aged between 23 and 41. A canula was inserted into the nucleus pulposa at the L4-5 level and connected to a pressure monitor using a pressure transducer. Distraction forces between 50 to 100 pounds were used. The author reported data on three of the five subjects. This was due to procedural difficulties associated with the first two subjects. Results showed decompression therapy reduced intradiscal pressure from -25 to -160 mm Hg. The author concluded additional study is needed to establish the relationship of negative intradiscal pressures with clinical outcomes.

#### Study limitations

It is difficult to base the physiologic effect of a treatment on a study of 5 subjects, especially when the results are only provided on three.

### The effects of vertebral axial decompression on sensory nerve dysfunction in patients with low back pain and radiculopathy [12]

This study tested the sensory nerve function on subjects with low back pain and radiculopathy. Seventeen patients were selected. A total of 22 nerves were tested due to multiple level involvement. The testing instrument used to measure outcomes was the Current Perception Threshold (CPT) Neurometer. Results of the study showed 64% returned to normal function, 27% improved and 4.5 % had no improvement and 4.5% showed deterioration. Patient outcomes were not measured in this study.

#### Study limitations

The primary concern with this paper is the outcome measure utilized. Aetna has issued a policy bulletin stating that "the effectiveness and clinical applicability of CPT testing in diagnosing or managing a disease has not been established"[13]. Additionally an American Academy of Neurology report concludes malingering and other non-organic factors can influence outcomes and this type of testing should not be used as a sole outcome measure [14].

### Sudden progression of lumbar disk protrusion during vertebral axial decompression traction therapy [15]

This was a case report of a 46 year old male with a three month history of radicular pain consistent with a S1 radiculopathy. During his 5^th ^session he suffered a severe exacerbation of his pain with marked enlargement of the disc protrusion requiring urgent microdiscectomy.

Decompression therapy has been marketed as completely safe. This case study demonstrates adverse events can occur.

In reviewing the literature many concerns were raised as to the objectivity of the published research. For example many of the studies performed utilized the VAX-D^® ^unit in which the patient position is prone [4,7,9-11]. Other manufacturers, although often referencing these studies in their advertising, have the patient in a supine position. This raises the question, is research valid for patient supine units when many of the studies were performed with the patient prone?

It appears that much of the research performed with decompression therapy is marketing oriented. Both of the Shealy studies were published in the "emerging technologies" section of the *American Journal of Pain Management*. This section is described by the journal editor as "either very small scale, uncontrolled, under-powered, and/or open-label. Studies under this heading should not be considered as standard, powered, blinded, controlled, cross-over designs". Two commonly quoted articles in the advertising of spinal decompression are found in a non peer-reviewed journal [16] or in "informational" sections of an internet newsletter[17]. A letter to the editor of the *Archives of Medical Rehabilitation*, in reference to a spinal decompression advertisement previously printed, stated "it appears this is a paid advertisement intentionally created in such a manner to deceive readers into believing that it is a true news story that the editors decided to publish for the information of its readers...all these components attempt to create the impression that it is an objective piece of medical journalism" [18].

An author in the only RCT of decompression therapy has a financial interest in VAX-D technology in Australia [4].

These observations raise concern as to the objectivity of the research for spinal decompression.

#### Limitations

Although the structure of this paper resembles a systematic review, it is not. It does not adhere to the strict requirements of a systematic review. The author did not address methods for each study or if the conclusions were accurate based on methods utilized. The individual studies were not graded according to an established grading system. The articles were simply reviewed and important shortcomings of the studies were reported. This paper was prepared by a single author and as a result might include bias although the author attempted to be fair in his assessment. This is a debate article. It is designed to initiate dialogue relating to the efficacy of non-surgical spinal decompression and as a result has methodological shortcomings.

## Summary

There is very limited evidence in the scientific literature to support the effectiveness of non-surgical spinal decompression therapy. This intervention has never been compared to exercise, spinal manipulation, standard medical care or other less expensive conservative treatment options which have an ample body of research demonstrating efficacy. Considering the cost-benefit relationship, many better researched and less expensive treatment options are available to the clinician.

## Competing interests

The author(s) declare that they have no competing interests.
